# Destructive Effects of Pyroptosis on Homeostasis of Neuron Survival Associated with the Dysfunctional BBB-Glymphatic System and Amyloid-Beta Accumulation after Cerebral Ischemia/Reperfusion in Rats

**DOI:** 10.1155/2021/4504363

**Published:** 2021-08-14

**Authors:** Zhongkuan Lyu, Yuanjin Chan, Qiyue Li, Qiang Zhang, Kaili Liu, Jun Xiang, Xiangting Li, Dingfang Cai, Yaming Li, Bing Wang, Zhonghai Yu

**Affiliations:** ^1^Geriatrics Department of Chinese Medicine, Huadong Hospital, Fudan University, Shanghai 200040, China; ^2^Department of Traditional Chinese Medicine, Shanghai Jiao Tong University Affiliated Sixth People's Hospital, Shanghai 200233, China; ^3^Department of Integrative Medicine, Zhongshan Hospital, Fudan University, Shanghai 200032, China

## Abstract

Neuroinflammation-related amyloid-beta peptide (A*β*) accumulation after cerebral ischemia/reperfusion (I/R) accounts for cerebral I/R injuries and poststroke dementia. Recently, pyroptosis, a proinflammatory cell death, has been identified as a crucial pathological link of cerebral I/R injuries. However, whether pyroptosis acts as a trigger of A*β* accumulation after cerebral I/R has not yet been demonstrated. Blood-brain barrier (BBB) and glymphatic system mediated by aquaporin-4 (AQP-4) on astrocytic endfeet are important pathways for the clearance of A*β* in the brain, and pyroptosis especially occurring in astrocytes after cerebral I/R potentially damages BBB integrity and glymphatic function and thus influences A*β* clearance and brain homeostasis. In present study, the method of middle cerebral artery occlusion/reperfusion (MCAO/R) was used for building models of focal cerebral I/R injuries in rats. Then, we used lipopolysaccharide and glycine as the agonist and inhibitor of pyroptosis, respectively, Western blotting for detections of pyroptosis, AQP-4, and A*β*_1-42_ oligomers, laser confocal microscopy for observations of pyroptosis and A*β* locations, and immunohistochemical stainings of SMI 71 (a specific marker for BBB integrity)/AQP-4 and Nissl staining for evaluating, respectively, BBB-glymphatic system and neuronal damage. The results showed that pyroptosis obviously promoted the loss of BBB integrity and AQP-4 polarization, brain edema, A*β* accumulation, and the formation of A*β*_1-42_ oligomers and thus increased neuronal damage after cerebral I/R. However, glycine could inhibit cerebral I/R-induced pyroptosis by alleviating cytomembrane damage and downregulating expression levels of cleaved caspase-11/1, N-terminal gasdermin D, NLRP3 (nucleotide-binding domain, leucine-rich repeat containing protein 3), interleukin-6 (IL-6) and IL-1*β* and markedly abate above pathological changes. Our study revealed that pyroptosis is a considerable factor causing toxic A*β* accumulation, dysfunctional BBB-glymphatic system, and neurological deficits after cerebral I/R, suggesting that targeting pyroptosis is a potential strategy for the prevention of ischemic stroke sequelae including dementia.

## 1. Introduction

Ischemic stroke, a common cerebrovascular disease, constitutes most strokes and is among the leading causes of long-term disability and dementia worldwide [1, 2]. For patients suffered from acute cerebral ischemia, there is a pressing need to restore blood flow of the ischemic cerebral tissue in a short-time window. However, the additional injuries following ischemia/reperfusion (I/R) greatly influence the therapeutic efficacy of restoring blood flow and induce ischemic stroke sequelae including dementia. As one of the key factors causing Alzheimer's disease (AD), neuroinflammation-related amyloid-beta peptide (A*β*) massively accumulates around astrocytes in ischemic brain tissues, accounting for cerebral I/R injuries and the occurrence of dementia induced by ischemic stroke [3–6].

Astrocytes in the brain are star-shaped cells with a range of functions, among which are to supply energy for neurons, underscoring the importance of astrocytes in neuroglial vascular coupling named neurovascular unit (NVU) [7]. Moreover, aquaporin-4 (AQP-4) on astrocytic endfeet functions as an important part of both blood-brain barrier (BBB) and glymphatic system to clear metabolic wastes such as A*β* and maintains the homeostasis of central nervous system (CNS) environment [8].

Pyroptosis is a newly discovered proinflammatory form of cell death distinguished from apoptosis, and it has been demonstrated that pyroptosis plays an important role during I/R injuries in multiple vital organs including the brain [9–11]. And the known signaling molecules involved in pyroptosis mainly include certain cysteine-dependent aspartate-directed proteases (caspase), proinflammatory factors such as interleukin-1*β* (IL-1*β*), nucleotide-binding oligomerization domain-like receptors pyrin domain containing 3 (NLRP3), and the gasdermin family [12]. Various studies have demonstrated that the gasdermin D (GSDMD) can be cleaved by activated caspase-4/5/11 (caspase-4/5 in humans, and the orthologous caspase-11 in rodents) or caspase-1 to form N-terminal (GSDMD-N) fragment which determines pyroptotic cell death by forming membrane pores [13–15].

Astrocytic and microglial pyroptosis induced by cerebral I/R has been reported [16, 17]. Therefore, we hypothesized and designed this study to identify that pyroptosis, especially occurring in astrocytes, aggravates the damage of BBB-glymphatic system, triggers the accumulation of toxic A*β*, and thus destroys homeostasis of neurons survival after cerebral I/R, and that inhibition of pyroptosis could ameliorate these pathological changes.

## 2. Materials and Methods

### 2.1. Animals

A total of sixty specific pathogen-free (SPF) male Sprague-Dawley rats, weighing 200-230 g, were obtained from Shanghai Laboratory Animal Research Center and housed in SPF animal rooms of Shanghai Jiao Tong University Affiliated Sixth People's Hospital, under the standard laboratory conditions with controlled humidity and constant temperature. Experimental operations were carried out after the acclimation of animals for several days with unlimited food and water. Both animal handling procedures and experimental protocols were consistent with the guidelines for the management of laboratory animals and approved by the Animal Ethics Committee of Shanghai Jiao Tong University Affiliated Sixth People's Hospital.

### 2.2. Groups and Interventions

Lipopolysaccharide (LPS) and glycine (Gly) are usually used as the activator and inhibitor of pyroptosis, respectively [18, 19]. Accordingly, the rats in this study were randomly divided into four groups including the sham group, cerebral ischemia/reperfusion group (I/R), cerebral ischemia/reperfusion plus LPS group (I/R + LPS), and cerebral ischemia/reperfusion plus Gly group (I/R + Gly). And LPS (125 *μ*g/ml) and Gly (200 mg/ml) (#L2630, #G7126, Sigma-Aldrich, USA) dissolved in distilled water were intraperitoneally injected (LPS, 500 *μ*g/kg; Gly, 800 mg/kg) at 3 h after cerebral I/R according to previous studies [20, 21]. Rats in other groups were injected with equivalent volume of distilled water.

### 2.3. Models of Focal Cerebral I/R Injuries and Neurological Function Assessment

The method of left middle cerebral artery occlusion/reperfusion (MCAO/R) was used for building models of focal cerebral I/R injuries in rats as described in our previous work [22, 23]. Rats in I/R, I/R + LPS, and I/R + Gly groups were subjected to MCAO/R surgeries, while rats in the sham group underwent the same operation with no insertion of the monofilament. Neurological examinations were performed after reperfusion. And in order to exclude the interference of operative failures, the rats subjected to MCAO/R with no detectable neurological deficits were eliminated from the following researches and analyses. During the whole course, rectal temperature and cardiovascular rate of all rats were monitored and maintained. Finally, eight rats were died or ruled out from the experiments. And neurological deficits score of rats at 24 h after reperfusion in present study were evaluated on a 5-point scale as described previously [24].

### 2.4. Brain Water Content Measurement

Brain water content was measured with the dry-wet weight method. Briefly, after being anesthetized with pentobarbital sodium (0.5%, 1 ml/100 g), the animals were sacrificed, and the brain tissues were removed and separated into ischemic and nonischemic hemispheres, which were immediately weighed to obtain the wet weight (WW). Then, the tissues were placed in an oven at 60°C for 24 h and reweighed to obtain the dry weight (DW). The brain water content was assessed with the following formula: 100% × (WW − DW)/WW.

### 2.5. Western Blotting Analysis for Detections of Pyroptosis, AQP-4, and A*β*_1-42_ Oligomers

After 24 h reperfusion, the rats were deeply anesthetized, and the brains were quickly removed after cardiac perfusion with 200 ml normal saline. The expression levels of pyroptosis-related proteins, AQP-4, and A*β*_1-42_ oligomers were detected by Western blotting. In brief, after concentrations measurement, equal amounts of protein extracted from ischemic penumbra and equivalent area under sham were separated by 10% sodium dodecyl sulfate-polyacrylamide gel electrophoresis (SDS-PAGE) and electrotransferred onto the polyvinylidnene fluoride membranes (#ISEQ00010, Millipore, USA). The membranes were blocked at room temperature with 5% bovine serum albumin (BSA) for 1 h and incubated with the following primary antibodies at 4°C overnight: caspase-11 (1 : 200, #sc-56038, Santa Cruz, USA), GSDMD (1 : 1000, #93709, Cell Signaling Technology, USA), NLRP3 (1 : 300, #19771-1-AP, Proteintech, USA), caspase-1 (1 : 500, #22915-1-AP, Proteintech), IL-6 (1 : 200, #sc-57315, Santa Cruz), IL-1*β* (1 : 200, #sc-12742, Santa Cruz), AQP-4 (1 : 200, #sc-32739, Santa Cruz), A*β*_1-42_ (1 : 1000, #ab201060, Abcam, UK), and *β*-actin (1 : 1000, #3700, Cell Signaling Technology). Then, the membranes were washed and incubated with corresponding secondary antibodies (1 : 5000, #L3012/L3032, Signalway Antibody, USA) for 1 h at room temperature. Western blotting bands were developed with the enhanced chemiluminescence kit (#WBKLS0500, Millipore), and then pictures were captured with a gel imaging instrument (Bio-Rad Laboratories, USA), and the intensities were analyzed by ImageJ software (National Institutes of Health, USA).

### 2.6. LDH Assay

Briefly, homogenates from cortex tissues in ischemic penumbra and equivalent area under sham were prepared and centrifuged at 1,2000 rpm and 4°C for 10 min, and then the supernatant was used to detect the content of LDH for preliminarily assessing the degree of pyroptosis by an LDH assay kit (#C0016, Beyotime, China) following the manufacturer's instructions.

### 2.7. Observations of Pyroptosis and A*β* Locations by Laser Confocal Microscopy

After anesthetization followed by infusion with normal saline and then 4% paraformaldehyde, the brains of rats were removed and immersed in 4% paraformaldehyde for 24 h fixation and then prepared for paraffin sections. According to the procedure described previously [25], we made an optimum proposal of PI staining in this study. Briefly, the paraffin sections were dewaxed and rehydrated (100% ethanol for 3 min, 95% ethanol for 2 min, 80% ethanol for 2 min, 75% ethanol for 2 min, H_2_O for 1 min) followed by incubation with PI dye (5 *μ*g/ml, #ST511, Beyotime) diluted by phosphate buffer solution (PBS) for 2 min at room temperature. Subsequently, PI dye was removed quickly and washed with PBS, and then 4′,6-diamidino-2-phenylindole (DAPI) staining solution was added onto the sections for 10 min at 37°C. For immunofluorescence stainings of protein colocalization, after dewaxing and rehydration with gradient ethanol, the sections further went through antigen retrieval, permeation by 0.3% triton-X 100, and then blockage with 5% BSA. Subsequently, the sections were incubated with the first antibodies mixed for caspase-11/ionized calcium-binding adapter molecule-1 (Iba-1) (#ab178847, Abcam), caspase-11/glial fibrillary acidic protein (GFAP) (#23935-1-AP, Proteintech), A*β* (#sc-28365, Santa Cruz)/GFAP, and GSDMD (#20770-1-AP, Proteintech)/GFAP (#60190-1-Ig, Proteintech) overnight at 4°C followed by incubations with corresponding mixed Alexa Fluor 488/647 secondary antibodies (1 : 500, #A0423/A0473, #A0428/A0468, Beyotime) for 1 h at room temperature. After DAPI staining, all the sections were covered with antiquenching agent for capturing fluorescent pictures by a laser scanning confocal microscope (Leica Wetzlar, Germany).

### 2.8. Evaluations of the BBB-Glymphatic System and Neuronal Damage

Endothelial barrier antigen (EBA, clone: SMI 71) is a specific marker for BBB integrity, and polarization loss of AQP-4 on astrocytic endfeet is the major cause of glymphatic dysfunction. Thus, we further made evaluations of BBB and glymphatic system by the immunohistochemical staining of SMI 71 and AQP-4. Briefly, the sections were dewaxed and rehydrated and went through antigen retrieval, permeation, and inactivation of the endogenous catalase by H_2_O_2_ and then blockage with 5% BSA. Subsequently, the sections were incubated, respectively, with anti-rat BBB antibody (SMI 71) (1 : 100, #836812, BioLegend, USA) and AQP-4 antibody (1 : 100, #sc-32739, Santa Cruz) overnight at 4°C followed by incubations with the secondary antibody in immunohistochemical kit (#KIHC-5, Proteintech) for 1 h at room temperature. Then, 3,3-diaminobenzidine tetrahydrochloride and hematoxylin were used as color developing reagents for visualizing the sections. Neuronal damage was evaluated by the method of Nissl staining as described in our previous study [22]. Briefly, the sections were dewaxed, rehydrated, and stained with Nissl staining solution (#E607316, Sangon Biotech, China) at room temperature for 20 min. Subsequently, the sections were rinsed and cleared in graded ethanol and xylene and coverslipped under permount. Finally, all sections were observed with a light microscope (Leica Wetzlar, Germany).

### 2.9. Statistical Analysis

All the data were expressed as the mean ± standard deviation (SD). Statistical analysis was performed using GraphPad Prism 8.0 (GraphPad Software Inc., USA). Statistical significance of difference among groups was analyzed by one-way ANOVA or unpaired Student's *t*-test. A value of *P* < 0.05 was considered to be statistically significant.

## 3. Results

### 3.1. Effects of LPS and Gly Interventions on Neurological Deficits

**Figure 1:**
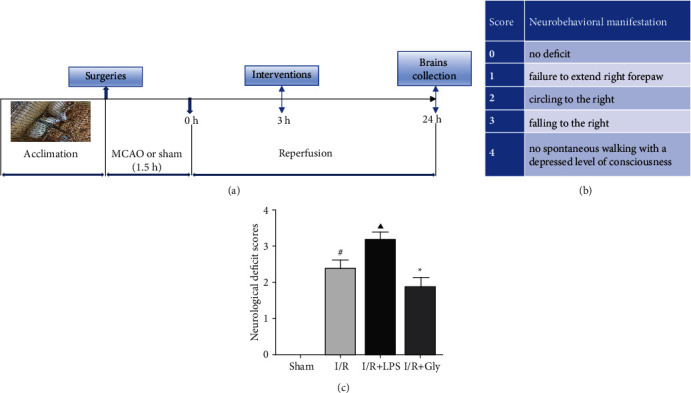
(a) Schematic diagram of the experimental protocol. (b) Longa's score and corresponding neurobehavioral manifestation. (c) Neurological deficit scores of each group according to Longa's score method, *n* = 10. Data are presented as mean ± SD.^#^*P* < 0.05, I/R group versus sham group; ^▲^*P* < 0.05, I/R + LPS group versus I/R group; ^∗^*P* < 0.05, I/R + Gly group versus I/R group.

Figure 1 exhibited the schematic diagram of experimental protocol (Figure 1(a)) and neurological deficits score of rats at 24 h after reperfusion (Figures 1(b) and 1(c)) in present study. The neurological deficits were evaluated on a 5-point scale as described in Figure 1(b). And the result of neurological function assessment showed that cerebral I/R-induced neurological deficits were obviously exacerbated by LPS but reversed by Gly as described in Figure 1(c).

### 3.2. Effects of LPS and Gly Interventions on Degree of Pyroptosis

**Figure 2:**
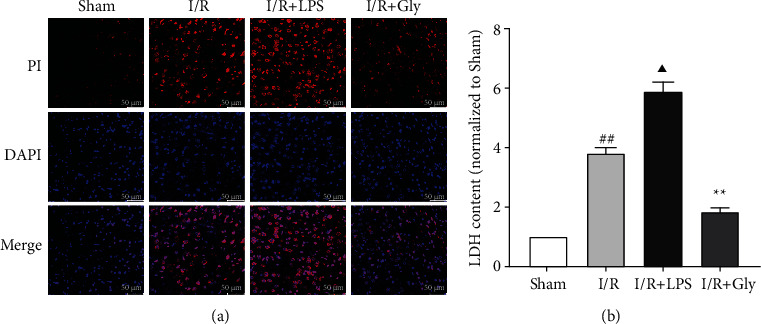
Effects of LPS and Gly interventions on the degree of pyroptosis at 24 h after reperfusion. (a) Representative pictures of PI staining, the red dots represent positive PI staining; scale bars, 50 *μ*m. (b) Relevant quantitative analysis of LDH content, *n* = 4. Data are presented as mean ± SD. ^##^*P* < 0.01, I/R group versus sham group; ^▲^*P* < 0.05, I/R + LPS group versus I/R group; ^∗∗^*P* < 0.01, I/R + Gly group versus I/R group.

Detection of the LDH content in damaged tissues and PI staining is effective methods used for preliminarily assessing the degree of pyroptosis. In present study, compared with the sham group, the I/R group showed higher LDH content and more positive PI staining which were obviously increased by LPS and reduced by Gly (Figures 2(a) and 2(b)), implying that pyroptosis was aggravated in the I/R + LPS group but alleviated in the I/R + Gly group after reperfusion.

### 3.3. Pyroptosis Focuses on Microglia and Astrocytes after Reperfusion

**Figure 3:**
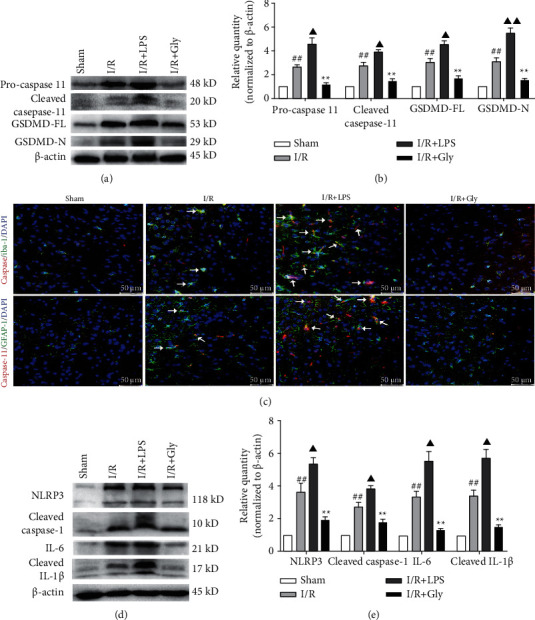
Effects of LPS and Gly interventions on expression levels of pyroptosis-related proteins and observations of pyroptosis locations. (a, b) Expression levels of pro-/cleaved caspase-11 and GSDMD-FL/N by Western blotting analysis, *n* = 6. (c) Representative pictures of the double immunofluorescence staining (white arrows) of caspase-11 (red) colocalized with Iba-1 or GFAP (green), respectively, scale bars, 50 *μ*m. (d, e) Expression levels of NLRP3, cleaved caspase-1, IL-6, and cleaved IL-1*β*, *n* = 6. Data are presented as mean ± SD. ^##^*P* < 0.01, I/R group versus sham group; ^▲^*P* < 0.05, ^▲▲^*P* < 0.01, I/R + LPS group versus I/R group; ^∗∗^*P* < 0.01, I/R + Gly group versus I/R group.

Pyroptosis is initially found as an innate immune response with strong inflammatory reaction. Thus, in this study, locations of pyroptosis were locked on the immunocyte in the brain. As expected, pyroptosis mainly occurred in microglia and astrocytes of ischemic brain tissues, which was observed from the double immunofluorescence staining of caspase-11 colocalized with Iba-1 (microglial biomarker) and GFAP (astrocytic biomarker), respectively, and was obviously aggravated in the I/R + LPS group but alleviated in the I/R + Gly group (Figure 3(c)). Correspondingly, the expression levels of pyroptosis-related proteins such as pro-/cleaved-caspase-11, GSDMD-FL (full length GSDMD)/N (Figures 3(a) and 3(b)), NLRP3, cleaved caspase-1, IL-6, and cleaved IL-1*β* (Figures 3(d) and 3(e)) significantly increased in the I/R + LPS group but decreased in the I/R + Gly group compared with those in the I/R group.

### 3.4. Pyroptosis Influences BBB Integrity after Reperfusion

**Figure 4:**
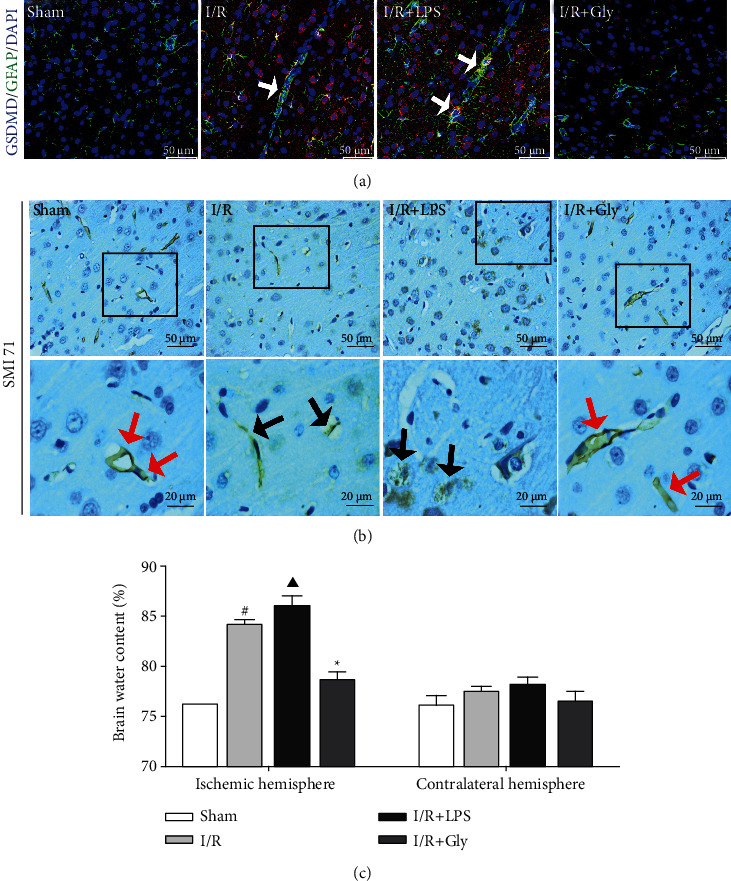
Influences of pyroptosis on BBB integrity at 24 h after reperfusion. (a) Representative pictures of double immunofluorescence stainings of GSDMD (red) colocalized with GFAP (green); white arrows represent astrocytic pyroptosis, scale bars, 50 *μ*m. (b) Representative pictures of immunohistochemical staining of SMI 71. Red arrows show smooth and intact capillaries which represent normal BBB integrity, and black arrows represent the damage of BBB integrity with unsmoothed, shriveled, or ruptured capillaries, scale bars, 50/20 *μ*m. (c) Brain water content analysis of ischemic hemisphere, *n* = 4. Data are presented as mean ± SD. ^#^*P* < 0.05, I/R group versus sham group; ^▲^*P* < 0.05, I/R + LPS group versus I/R group; ^∗^*P* < 0.05, I/R + Gly group versus I/R group.

Astrocytic endfeet envelops the cerebral capillaries that form BBB. Our study showed that the damage of BBB integrity reflected by immunohistochemical staining of SMI 71 was worsened with the exacerbation of astrocytic pyroptosis in the I/R + LPS group but alleviated with the mitigation of astrocytic pyroptosis in the I/R + Gly group, respectively (Figures 4(a) and 4(b)). Accordingly, the water content of ischemic hemisphere significantly increased in the I/R + LPS group but decreased in the I/R + Gly group compared with that in the I/R group (Figure 4(c)).

### 3.5. Pyroptosis Influences AQP-4 Polarization and A*β* Clearance after Reperfusion

**Figure 5:**
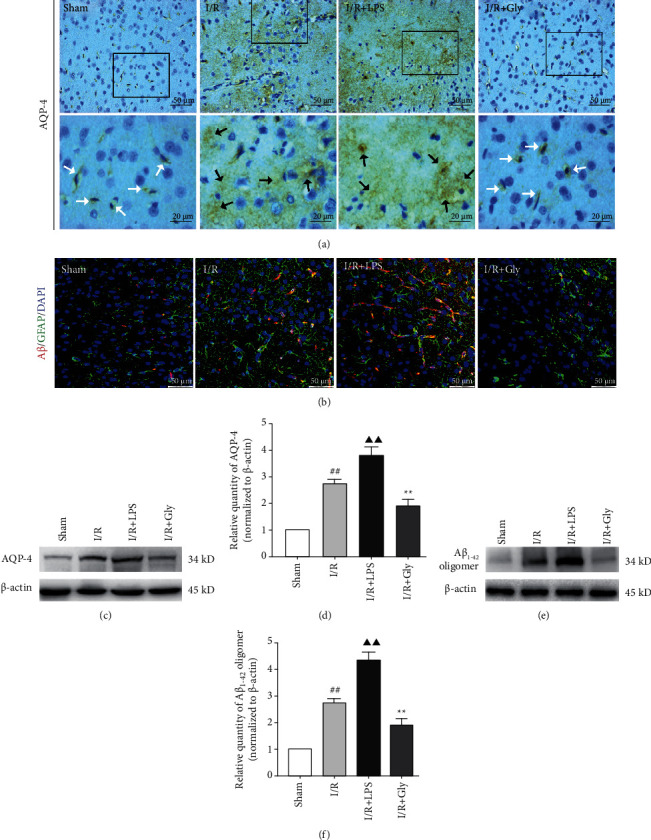
Influences of pyroptosis on AQP-4 polarization and A*β* clearance at 24 h after reperfusion. (a) Representative pictures of immunohistochemical staining of AQP-4. White arrows represent normal AQP-4 polarization, and black arrows represent the loss of AQP-4 polarization with obvious dispersion and perturbed expression, scale bars, 50/20 *μ*m. (b) Representative pictures of double immunofluorescence staining of A*β* (red) colocalized with GFAP (green), scale bars, 50 *μ*m. (c, d) Protein levels of AQP-4 by Western blotting analysis, *n* = 6. (e, f) Protein levels of A*β*_1-42_ oligomer by Western blotting analysis, *n* = 6. Data are presented as mean ± SD. ^##^*P* < 0.01, I/R group versus sham group; ^▲▲^*P* < 0.01, I/R + LPS group versus I/R group; ^∗∗^*P* < 0.01, I/R + Gly group versus I/R group.

The AQP-4-dependent glymphatic system is an important pathway for the clearance of A*β* in the brain. And glymphatic dysfunction is closely associated with the loss of AQP-4 polarization on astrocytic endfeet. In present study, the results showed that the loss of AQP-4 polarization after reperfusion with obvious dispersion and perturbed expression was worsened in the I/R + LPS group but apparently lightened in the I/R + Gly group (Figures 5(a), 5(c), and 5(d)). Accordingly, the accumulation of A*β* concentrated around astrocytes was also aggravated in the I/R + LPS group but alleviated in the I/R + Gly group (Figure 5(b)). Furthermore, A*β*_1-42_ oligomer (the main form of toxic A*β*) obviously increased in the I/R + LPS group but decreased in the I/R + Gly group compared with that in the I/R group (Figures 5(e) and 5(f)).

### 3.6. Pyroptosis Influences Neuron Survival after Reperfusion

**Figure 6:**
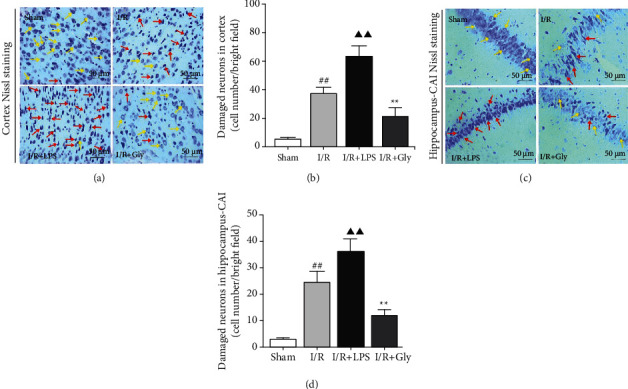
Influences of pyroptosis on neurons survival at 24 h after reperfusion. (a, c) Representative pictures of ischemic cortex and hippocampus-CA1 Nissl staining. Yellow arrows represent normal morphology of neurons with clear nucleolus, abundant cytoplasm, and intact structure. Red arrows represent abnormal neurons appeared shrunken and deep stained, scale bars, 50 *μ*m. (b, d) Quantitative analysis of damaged neurons in the cortex and hippocampus-CA1 areas, *n* = 6. Data are presented as mean ± SD. ^##^*P* < 0.01, I/R group versus sham group; ^▲▲^*P* < 0.01, I/R + LPS group versus I/R group; ^∗∗^*P* < 0.01, I/R + Gly group versus I/R group.

**Figure 7:**
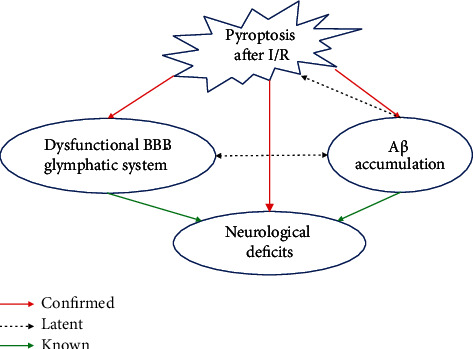
Schematic diagram about internal relationships among pathological changes in this study. Red arrows present relationships confirmed by this study among pathological changes including pyroptosis, A*β* accumulation, dysfunctional BBB-glymphatic system, and neurological deficits after I/R, and dotted arrows and green arrows, respectively, present latent and known relationships obtained on the basis of previous studies.

The above results in present study have revealed that cerebral I/R-induced pyroptosis promotes dysfunctions of the BBB-glymphatic system and toxic A*β* accumulation and thus potentially influences the CNS homeostasis on which neurons survival depend. Accordingly, our study further showed that the damaged neurons in ischemic cortex (Figures 6(a) and 6(b)) and hippocampus (Figures 6(c) and 6(d)) tissues after reperfusion significantly increased in the I/R + LPS group but decreased in the I/R + Gly group compared with those in the I/R group, which was consistent with the results of neurological function assessment. Furthermore, Figure 7 summarizes internal relationships of pyroptosis promoting neurological deficits associated with the dysfunctional BBB-glymphatic system and A*β* accumulation after I/R in this study, which would be expatiated detailedly in the following discussion.

## 4. Discussion

Damage of cell membrane is a common pathological change of I/R injuries [26, 27]. Cellular membrane permeabilization is a prominent feature of pyroptosis, and the GSDMD in the gasdermin family is identified as the key executor of pyroptosis to damage the integrity of cellular membranes by forming nanopores which cause cellular swelling and death [13–15]. Therefore, recently, the roles of pyroptosis and its related signaling molecules such as caspase-11/1 and GSDMD in I/R injuries have been attracting attention of researchers [9–11]. LPS is the common agonist of pyroptosis by activating caspase-11, and Gly is usually used as the protective agent of cellular membrane to inhibit pyroptosis [18, 19]. It has been demonstrated that pyroptosis is an important pathological link of cerebral I/R injuries [11], and our results in present study showed that the pyroptosis-related damage of cell membrane after cerebral I/R could be, respectively, aggravated and alleviated by LPS and Gly interventions.

Previous literatures have indicated that the summit of cerebral I/R induced pyroptosis occurs at 24 h after reperfusion [11]. Our study showed that both microglia and astrocytes are the main locations of noncanonical pyroptosis mediated by caspase-11 which could trigger the cleavage of GSDMD and then the activation of NLRP3/caspase-1 pathway, causing acute neuroinflammation at 24 h after cerebral I/R along with the release of proinflammatory factors such as IL-1*β* and IL-6. Astrocytic endfeet envelops the cerebral capillaries that form BBB to exert the transport function of nutrition or metabolic products [28], and BBB dysfunction in I/R injuries is closely related to abnormal astrocytes which cause brain edema formation and nonreflow phenomenon and promote neuronal damage, greatly influencing effects of restoring blood flow [29–31]. Therefore, astrocytic pyroptosis was specially highlighted and exhibited in this study; though, we noticed that pyroptosis may occur in a few neurons or other brain cells besides microglia and astrocytes which could be observed by the result of GSDMD staining after I/R. Furthermore, our study indicated that aggravating pyroptosis promotes the damage and dysfunction of BBB, and inhibiting pyroptosis by Gly could abate the damage of BBB and significantly reduce brain edema after reperfusion.

Primary Gly at low level is an inhibitory neurotransmitter in CNS. However, interestingly, Gly at high level (800 mg/kg, intraperitoneal administration) inversely exerts protective effects against neuronal injury induced by I/R in rats according to the previous study [21]. Therefore, our current study adopted such level (800 mg/kg) of Gly as the intervention dosage administrated to rats, and we preliminarily observed the equal neuroprotective effect of Gly by assessing neurological function deficits. Then, our results further confirmed the inhibitory effects of Gly on pyroptosis after I/R. Multiple studies have demonstrated that Gly exerts cytoprotection against pyroptosis by targeting plasma membrane permeability barriers [19]. However, in current study, the results showed that Gly also exerts inhibitory effects on the expression levels of pyroptosis-related molecules such as cleaved caspase-11/1, N-terminal gasdermin D, and IL-1*β*. It is firstly considered that the protective effects of Gly on plasma membrane permeability barriers prevent the stimulators including abnormal iron current from aggravating pyroptosis in the setting of this study and thus mediately downregulate the expressions of pyroptosis-related signaling molecules. In addition, this phenomenon is also likely associated with the complex interrelated processes of pyroptosis. Recent evidence has revealed that IL-1*β* reversely induces the expression of caspase-11 during sterile inflammation [32]. Therefore, acting on one link tends to cause changes of its upstream or downstream processes during pyroptosis, which needs to be further exploration in specific experimental settings.

Recently, researches have demonstrated that toxic A*β*, one of crucial damage-associated molecular patterns (DAMPs) in AD, accumulates in brain and is responsible for brain edema formation and the occurrence of dementia induced by ischemic stroke and thus maintaining the clearance of A*β* after stroke could offer a new therapeutic approach to prevent poststroke cognitive impairment and development into dementia [5, 6, 33]. Numerous studies revealed that neuroinflammation is the fundamental factor triggering the generation of A*β* [34–36], and the accumulation of toxic A*β* is not only the outcome of BBB dysfunction but also the important cause of BBB damage [33, 37], just as our previous researches indicated that A*β*_1-42_ oligomers were the main form of A*β* toxicity inducing dysfunctional BBB [38, 39]. In this study, our results showed that cerebral I/R induces the concentration of A*β* around astrocytes and the formation of massive A*β*_1-42_ oligomers within 24 h after reperfusion, and that aggravating pyroptosis obviously increases A*β*_1-42_ oligomers while inhibiting pyroptosis by Gly could markedly reverse A*β* accumulation and the formation of A*β*_1-42_ oligomers. Therefore, pyroptosis acts as a considerable trigger of toxic A*β* accumulation after cerebral I/R.

The glymphatic system is an important pathway besides BBB for the clearance of A*β* in the brain [33]. AQP-4, the main component of glymphatic system, is a water channel physiologically located with high polarization on astrocytic endfeet, and the loss of AQP-4 polarization can cause the dysfunction of glymphatic system in pathological changes including AD and ischemic stroke [40, 41]. Thus, AQP-4 is now recognized as essential for two unique functions, namely, neurovascular coupling and glymphatic flow, to facilitate the clearance of metabolic wastes such as A*β* [41, 42]. In present study, we observed that cerebral I/R-induced AQP-4 polarization loss with obvious dispersion and perturbed expression and A*β* concentration around astrocytes along with increased A*β*_1-42_ oligomers were exacerbated by the further activation of pyroptosis but obviously abated by the inhibition of pyroptosis, indicating that pyroptosis is an important factor causing glymphatic dysfunction which accounts for the accumulation of A*β*.

Previous researches have revealed that chronic A*β* accumulation around astrocytes accompanies with not only BBB damage but delayed neuronal death (DND) within six months after cerebral I/R and even deposits to plaques with the further extension of time [43, 44], which provide experimental evidence for the occurrence of sporadic AD induced by ischemic stroke. Furthermore, a clinical study by Liu et al. [5] demonstrated that patients with A*β* deposition experienced a more severe and rapid cognitive decline over 3 years after stroke/transient ischemic attack compared with subjects without AD-like A*β* deposition, and A*β* was associated with changes in multiple cognitive domains. Recently, Martins et al. [6] have reported the acute accumulation of A*β* oligomers within 24 h in blood vessel walls including small capillaries and nearby brain tissues after cerebral I/R, and that such accumulation acts as a detrimental factor promoting brain damage. Additionally, the accumulation of toxic A*β* can produce swelling in astrocytes and their endfeet and also cause dysregulation of capillaries by acting on pericytes, influencing energy supply for neurons [45, 46]. Our study revealed that pyroptosis accounts for dysfunctions of the BBB-glymphatic system and the accumulation of toxic A*β* and thus destroys the CNS homeostasis on which neurons survival depend, aggravating the damage of neurons and neurological deficits after cerebral I/R. However, inhibiting pyroptosis could markedly abate these pathological changes. On the other hand, toxic A*β* has been identified as a trigger of pyroptosis and neuroinflammation [47, 48], which suggests a magnified effect of A*β* accumulation after cerebral I/R.

In the light of the above discussion, the outcome in present study firstly confirmed that cerebral I/R-induced pyroptosis significantly promotes the dysfunctional BBB-glymphatic system, A*β* accumulation, and the formation of toxic A*β* which have been known as crucial pathological factors influencing the CNS homeostasis on which neurons survival depend and thus increasing neurological deficits. Besides, after cerebral I/R, there exists a latent relationship with mutual stimulation between the dysfunctional BBB-glymphatic system and A*β* accumulation based on previous studies, and toxic A*β* potentially aggravating pyroptosis also deserves to be further demonstrated. Admittedly, the limitation of our current study may be that glycine could not exert a completely absolute blocking effect on pyroptosis of CNS. To solve this problem, we will further construct GSDMD gene knockout rats to indepth study pyroptosis and related pathological changes after cerebral I/R. Furthermore, we speculate and will confirm in the following study that pyroptosis and the acute accumulation of A*β* form a vicious circle in cerebral I/R injuries, which triggers the chronic A*β* accumulation after reperfusion and induces poststroke cognitive impairment.

## 5. Conclusion

The present study demonstrated that pyroptosis is a considerable factor causing the dysfunctional BBB-glymphatic system and the accumulation of toxic A*β* (A*β*_1-42_ oligomers) and thus aggravating the neuronal damage and neurological deficits after cerebral I/R in rats. And our study not only further identifies pyroptosis as an important link in cerebral I/R injuries but also suggests that targeting pyroptosis is a potential strategy for the prevention of ischemic stroke sequelae including dementia.

## Data Availability

The data used to support the findings of this study are available from the corresponding author upon request.
